# Coherent control of light-induced torque on four-level tripod atom systems

**DOI:** 10.1038/s41598-023-38866-4

**Published:** 2023-07-20

**Authors:** Ali Mehdinejad

**Affiliations:** Department of Physics, Sharif University of Technology, Tehran, Iran

**Keywords:** Optics and photonics, Physics

## Abstract

This paper investigates the manipulation of induced torque on a four-level tripod atom system through the interaction with two vortex probe beams featuring spatial inhomogeneity, along with a non-vortex control beam. The study explores both the linear and nonlinear regimes of torque induction. In the linear regime, where the intensity of the vortex beams is weaker than that of the control field, effective control over the induced torque is demonstrated by adjusting parameters such as magnetic field strength, control field intensity, detuning, and dephasing terms between relevant atomic levels. The analysis highlights the significant contribution of the Zeeman shift-induced magnetic field, which enhances the torque and exhibits a distinct sharp peak. Furthermore, higher-order contributions to the induced torque are discussed as the intensity of the probe fields approaches that of the control field, resulting in further enhancement of the induced torque. These findings offer opportunities for precise control over the rotational motion of atoms within the system, with potential applications in precision measurement, quantum information processing, and quantum control.

## Introduction

The fascinating realm of modern nonlinear and quantum optics revolves around harnessing the remarkable principles of quantum interference and coherence to manipulate and reshape the characteristics of both light and matter. Within the domain of multilevel atomic systems, the interplay of quantum coherence and interference gives rise to a plethora of captivating optical phenomena. These include extraordinary phenomena like lasing without the need for population inversion (LWI)^1–4^, the ability to amplify light without introducing absorption^5,6^, the utilization of stimulated Raman adiabatic passage (STIRAP)^7,8^, the augmentation of Kerr nonlinearity, the manifestation of optical bistability^9–11^, and the phenomenon of electromagnetically induced transparency (EIT)^12–14^, among others.

Researchers have delved into the concept of LWI, a groundbreaking achievement in the field. In this intriguing phenomenon, the typical requirement of an inverted population of energy levels is circumvented, enabling laser emission under unconventional conditions. Enhanced index of refraction without absorption, an exciting prospect challenges traditional assumptions by allowing the manipulation of refractive properties without introducing unwanted energy loss. This breakthrough holds significant potential for various applications demanding precise control over light propagation. The STIRAP offers a powerful technique for transferring population between quantum states. By skillfully manipulating the system’s energy levels and laser pulses, one can achieve efficient and precise population transfer, opening avenues for advanced quantum control. The optical bistability and Kerr nonlinearity hold promise for shaping and manipulating light in nonlinear optical devices. By enhancing the nonlinear response of a material, novel applications in areas such as optical logic gates, all-optical switching and information processing become attainable. The EIT presents an enthralling effect where a normally opaque medium becomes transparent due to quantum interference. This phenomenon paves the way for further advancements, especially in slow light^15–17^.

Light beams possessing spiral phase dislocations exhibit a fascinating property known as orbital angular momentum (OAM), which stems from the peculiar phase distribution across their cross-section^18,19^. These beams exhibit a spiral phase characterized by the expression $e^{il\phi }$, where the phase is directly proportional to the azimuthal angle $\phi$. At the center of the beam, a null field is present, accentuating the distinctive properties of the OAM. The OAM carried by these light beams is quantized, taking on integer values denoted by the symbol *l*. This integer, referred to as the topological charge or beam order, serves as a measure of the number of azimuthal $2\pi$ phase shifts undergone by the beam^18^. Consequently, the presence of a phase singularity at the beam’s core gives rise to a ring-shaped intensity profile, imparting a distinct visual appearance to the beam. Over the years, researchers have successfully generated optical vortices characterized by phase singularities^20^. These vortices are now routinely created with the ability to carry specific values of OAM, thereby enabling precise manipulation and control of the angular momentum properties of light beams^21^.

The interaction between optical vortices and matter gives rise to a diverse array of intriguing effects, as extensively studied by researchers^22–32^. Among these effects, particular attention has been devoted to exploring the mechanical consequences of orbital angular momentum (OAM) on particles and atoms^33^. Notably, light beams possessing OAM have been effectively employed to induce rotation in particles confined within optical tweezers^34,35^. The transfer of OAM to matter has been shown to engender a torque linked to the transfer of wave energy^36–39^.

Andersen et al. demonstrated a remarkable phenomenon involving the coherent transfer of photon OAM to a Bose-Einstein condensate (BEC) composed of sodium atoms. This was achieved through a two-photon stimulated Raman process employing Laguerre-Gaussian (LG) beams^38^. By employing counter-propagating LG and Gaussian laser beams with the same linear polarization, the BEC was effectively trapped axially, and the OAM of the LG beam induced a torque on the center of mass of each trapped atom, causing the entire medium to rotate around the beam axis and resulting in the formation of an atomic vortex state.

In an alternative study, Lembessis and Babiker proposed a different scenario for inducing torque on a three-level $\Lambda$ BEC gas, where two counter-propagating spatially-dependent beams interacted with the atoms, resulting in a current flow^40^. This torque exerted a rotational effect on the entire BEC, as the many-body wave function of the BEC could be approximated by the product of identical single-particle wave functions^38^. Very recently, Hamedi et al. investigated the light-induced enhanced torque on a double-V-type quantum emitter through quantum interference in spontaneous emission^41^. The magnitude of this induced torque was found to be controllable through the initial internal state preparation of the emitter. Additionally, they examined a fascinating scenario wherein the double-V emitter was positioned above the surface of a bismuth-chalcogenide material, while vortex beams propagated parallel to the surface. Numerical investigations were conducted to explore the consequences of spontaneous emission interference induced by the presence of bismuth-chalcogenide microstructures on the imposed torque experienced by the double-V emitters.

These investigations shed light on the captivating interplay between optical vortices and matter, revealing the profound influence of OAM on the rotational dynamics of particles and atoms. The manipulation of induced torque on atoms indeed holds significant importance in specific experiments aimed at characterizing the dynamics of the ensemble. Moreover, the proposed torque on tripod atoms has promising applications in the realization of superconducting atomic devices. By harnessing the induced torque, it becomes possible to generate superflow and achieve the superposition of macroscopic states in atomic vapors^42^. This paves the way for advancements in superconducting atomic devices and opens up possibilities for studying quantum phenomena on a macroscopic scale. Additionally, the manipulation of induced torque on tripod atoms finds utility in the field of quantum repeaters. Quantum repeaters play a crucial role in long-distance quantum communication, where the challenge lies in preserving the delicate quantum information over extended transmission distances. By utilizing the induced torque and the associated OAM-carrying photons, quantum repeaters can be designed to enhance the transmission and storage of quantum information^43^. This has the potential to revolutionize secure quantum communication networks by enabling efficient and robust long-distance quantum information transfer. The proposed torque on tripod atoms not only holds promise for precision measurement and quantum information processing, but also finds utility in superconducting atomic devices and quantum repeaters. These applications showcase the versatility and potential impact of the proposed torque in various fields of research and technology. With this inspiration, this paper investigates the induced torque on a four-level tripod atom system. The system is subjected to two counter-propagating, spatially inhomogeneous vortex probe beams, along with a non-vortex control beam. In the linear regime, the effective control over the system’s torque can be achieved by adjusting various factors such as magnetic field strength, intensity and detuning of the control field, and the dephasing terms between relevant atomic levels. Notably, we observe that the magnetic field resulting from the Zeeman shift of atom levels plays a crucial role in enhancing the torque. This leads to spike-like behavior in the torque as a function of probe detuning, offering precise control over the rotational motion of the atoms within the system. Such a torque is shown to further enhanced in the nonlinear regime, when the intensity of the probe fields is comparable with the control field. Our findings highlight the potential for manipulating induced torque in these systems, presenting opportunities in precision measurement, quantum information processing, and quantum control.

The four-level tripod scheme presents clear advantages over the three-level $\Lambda$^40^ or four-level double-V^41^ schemes, particularly in achieving a larger induced torque. These advantages stem from the inclusion of additional levels and transitions, which enhance the system’s interaction with external fields. Compared to the three-level $\Lambda$ scheme, the four-level tripod scheme introduces an extra laser field and atomic level, resulting in distinct peaks at different positions of probe detuning. This leads to diverse shapes for the induced torque, providing greater flexibility in tailoring the rotational motion of the system. In contrast, the three-level $\Lambda$ scheme does not possess this capability. Additionally, the four-level tripod scheme offers enhanced control over the system’s rotational motion by manipulating crucial parameters such as magnetic field strength, probe detuning, and control field intensity or detuning. This level of control was notably absent in a recent study by Hamedi et al.^41^. By incorporating these control mechanisms, our study expands the possibilities for manipulating the induced torque, enabling precise control over the rotational motion of atoms within the system. Moreover, our study goes beyond the linear regime and explores the nonlinear regime, where the intensities of the probe and control fields are comparable and exhibit strong interactions. In contrast, the analysis conducted in the previous study^41^ focused solely on the weak light-matter interaction regime. By considering the nonlinear regime, our study enhances the control over the system’s rotational motion, which proves advantageous for applications such as precision measurement or quantum information processing.

## Model and equations

**Figure 1:**
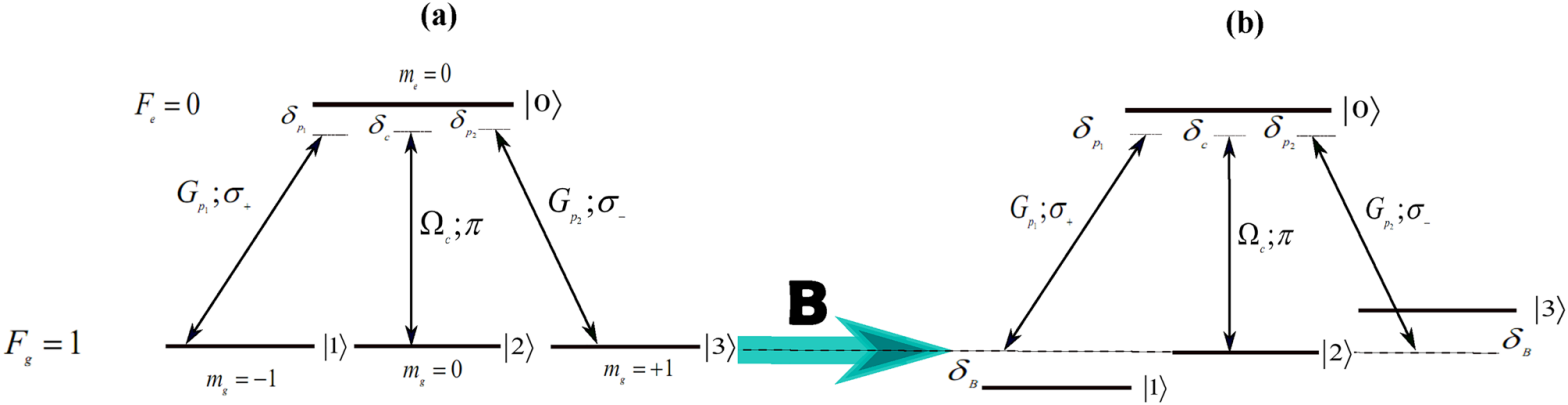
(**a**) The degenerate $F_{g}=1\rightarrow F_{e}=0$ transition is shown interacting with symmetrically detuned $\sigma _{+}$- and $\sigma _{-}$-polarized probes and a $\pi$-polarized control field. (**b**) The $F_{g}=1\rightarrow F_{e}=0$ transition is shown with its degeneracy lifted by applying a magnetic field ${\varvec{B}}$. This transition is interacting with a single $\sigma _{-}$-polarized probe and a $\pi$-polarized control field.

We investigate a tripod system that includes an excited level and three lower levels. The system interacts with two weak probe lasers that have equal Rabi frequencies, $G_{p_{1}}$ and $G_{p_{2}}$, and a strong control laser of Rabi frequency $\Omega _{c}$. The tripod configuration can be created by interacting a tunable, $\pi$-polarized strong control field (with a carrier frequency of $\omega _{c}$) with an $F_{g}=1\rightarrow F_{e}=0$ atomic transition. This transition is driven by symmetrically detuned $\sigma _{+}$ and $\sigma _{-}$ polarized probe beams (with carrier frequencies $\omega _{p_{1}}$, $\omega _{p_{2}}$) as shown in Fig. 1a. Another way to achieve a similar setup is by lifting the degeneracy of the $F_{g}=1$ state using a weak magnetic field and driving the system with a single $\sigma$-polarized pump at the resonance frequency of the degenerate $F_{g}=1\rightarrow F_{e}=0$ transition, as depicted in Fig. 1b. The tripod system, which is nearly degenerate, is ideal for high-resolution spectroscopy. Moreover, the configuration in Fig. 1b can be used as a magneto-optic switch to measure small magnetic fields. Furthermore, it enables sub-Doppler and subnatural narrowing of an absorption line through the interaction of dark resonances^44^. Additionally, it facilitates the occurrence of polarization phase gates, which have significant implications in various applications^45^.

Assuming the dipole approximation and the rotating wave approximation, the tripod Hamiltonian can be expressed in the interaction picture as follows:

1 $$\begin{aligned} H_{int}&=\hbar \left( \delta _{p_{1}}+\delta _{B}\right) \sigma _{00} +\hbar \left( \delta _{p_{1}}+\delta _{B}-\delta _{c}\right) \sigma _{22} +\hbar \left( \delta _{p_{1}}-\delta _{p_{2}}+2\delta _{B}\right) \sigma _{33}\nonumber \\&\quad + \hbar \left( G_{p_{1}}^{*}\sigma _{10}+G_{p_{1}}\sigma _{01} +\Omega _{c}^{*}\sigma _{20}+\Omega _{c}\sigma _{02}+G_{p_{2}}^{*}\sigma _{30} +G_{p_{2}}\sigma _{03}\right) , \end{aligned}$$

where $\sigma _{xy}=|x\rangle \langle y|$ are pseudospin atomic operators. Here, $\delta _{p_{1}}=\omega _{01}-\omega _{p_{1}}$ represents the detuning of the first probe laser from its corresponding transition, $\delta _{p_{2}}=\omega _{03}-\omega _{p_{2}}$ represents the detuning of the second probe laser, and $\delta _{c}=\omega _{02}-\omega _{c}$ represents the detuning of the control laser. In the absence of the fields, the population is evenly distributed amongst the Zeeman ground sublevels. The Zeeman shift of levels $|1\rangle$ and $|3\rangle$ is given by $\hbar \delta _{B}=\mu _{B}m_{s}g_{s}B$, where $\mu _{B}$ is the Bohr magneton, $g_{s}$ is the Lande factor, and $m_{s}=\pm 1$ is the magnetic quantum number of the corresponding sublevel. At this stage, spontaneous emission and dephasing are not considered, but they will be included later.

The electric dipole approximation allows the expression of the vortex beams $G_{p_{1}}$ and $G_{p_{2}}$ as:

2 $$\begin{aligned} G_{p_{1}}&=\Omega _{p_{1}}e^{i\eta _{1}({{\textbf {R}}})}, \end{aligned}$$

3 $$\begin{aligned} G_{p_{2}}&=\Omega _{p_{2}}e^{il\eta _{2}({{\textbf {R}}})}. \end{aligned}$$

The phase functions associated with the light beams are denoted as $\eta _{1}({{\textbf {R}}})=-l\phi +kz$, $\eta _{2}({{\textbf {R}}})=-l\phi -kz$, while $\Omega _{p_{1}}$ and $\Omega _{p_{2}}$ represent the Rabi frequencies. For doughnut Laguerre-Gaussian (LG) beams exhibiting the same spatial dependence, these Rabi frequencies can be expressed as follows:

4 $$\begin{aligned} \Omega _{p_{1}}&=g_{p_{1}}\left( \frac{r}{w}\right) ^{|l|}e^{-r^{2}/w^{2}}, \end{aligned}$$

5 $$\begin{aligned} \Omega _{p_{2}}&=g_{p_{2}}\left( \frac{r}{w}\right) ^{|l|}e^{-r^{2}/w^{2}}. \end{aligned}$$

In this context, the cylindrical radius is denoted by *r*, the topological (OAM) number is represented by an integer *l*, and the azimuthal angle is given by $\phi$. The beam waist is characterized by w, while $g_{p_{1}}$ and $g_{p_{2}}$ represent the strengths of the position-dependent beams. It should be noted that the effects of interest occur near the beam waist, which is located in the $z=0$ plane.

Assuming that the trapped atoms can be described by identical single-particle wave functions , the many-body wave function can be approximated as their product. In this case, each atom experiences the same force due to the light, and the single-body wave function can be expressed as a product of internal and center-of-mass wave functions^41^. The scattering force acting on the center-of-mass coordinate ${{\textbf {R}}}$ only involves density matrix elements of the internal states:

6 $$\begin{aligned} {{\textbf {F}}}=2\hbar \left[ \nabla \eta _{1}({{\textbf {R}}})\Omega _{p_{1}}(\rho _{10}+\rho _{01}) +\nabla \eta _{2}({{\textbf {R}}})\Omega _{p_{2}}(\rho _{30}+\rho _{03})\right] , \end{aligned}$$

where the coherences $\rho _{10}$ and $\rho _{30}$ are density matrix elements associated with the probe vortex transitions.

Using Eqs. (4)–(6) one can obtain the expression for the force:

7 $$\begin{aligned} {{\textbf {F}}}=2\hbar k\psi \left( \frac{r}{w}\right) ^{2|l|}e^{-2r^{2}/w^{2}}{\hat{z}}+\frac{2\hbar l}{r}\tau \left( \frac{r}{w}\right) ^{2|l|}e^{-2r^{2}/w^{2}}{\hat{\phi }}, \end{aligned}$$

where

8 $$\begin{aligned} \psi =\Omega _{p_{2}}\left( \rho _{30}+\rho _{03}\right) -\Omega _{p_{1}}\left( \rho _{10}+\rho _{01}\right) , \end{aligned}$$

9 $$\begin{aligned} \tau =-\Omega _{p_{1}}\left( \rho _{10}+\rho _{01}\right) -\Omega _{p_{2}}\left( \rho _{30}+\rho _{03}\right) . \end{aligned}$$

It is important to note that in Eq. (7), the symbols with carets represent unit vectors in cylindrical coordinates. We can express the torque exerted on the center of mass of the tripod atoms about the beam axis as:

10 $$\begin{aligned} {{\textbf {T}}}={{\textbf {r}}}\times {{\textbf {F}}}=2\hbar l \left( \frac{r}{w}\right) ^{2|l|}e^{-2r^{2}/w^{2}}\tau \,{\hat{z}}, \end{aligned}$$

indicating the induced torque has a quantized nature, increasing with larger topological numbers. The torque has a doughnut-shaped profile with a region of maximum intensity, forming an optical dipole potential trap that attracts the tripod atoms. The magnitude of the torque depends on the system’s controlling parameters, which are incorporated in the density matrix elements $\rho _{10}$ and $\rho _{30}$ that enter the function $\tau$. This feature allows for the control and enhancement of the torque, leading to an intensity-controllable ring-shaped flow. In the following section, we will derive analytical solutions for $\rho _{10}$ and$\rho _{30}$, in both linear and nonlinear regimes, and investigate how the system parameters affect the induced torque’s variation.

To provide a complete understanding of the dynamics of the four-level tripod model, we use the Bloch equations for the density matrix elements, which take into account atomic spontaneous emission and dephasing.

11 $$\begin{aligned} {\dot{\rho }}_{00}\,&=-\sum _{j=1}^{3}\gamma _{jj}\rho _{00}-i\Omega _{p_{1}}^{*}\rho _{10} +i\Omega _{p_{1}}\rho _{01}-i\Omega _{c}^{*}\rho _{20}+i\Omega _{c}\rho _{02} -i\Omega _{p_{2}}^{*}\rho _{30}+i\Omega _{p_{2}}\rho _{03}, \end{aligned}$$

12 $$\begin{aligned} {\dot{\rho }}_{11}&=\gamma _{11}\rho _{00}+\sum _{k=2,3}\gamma _{1k}\rho _{kk}-i\Omega _{p_{1}}\rho _{01}+i\Omega _{p_{1}}^{*}\rho _{10}, \end{aligned}$$

13 $$\begin{aligned} {\dot{\rho }}_{22}&=\gamma _{22}\rho _{00}-\gamma _{12}\rho _{22}+\gamma _{23}\rho _{33}-i\Omega _{c}\rho _{02}+i\Omega _{c}^{*}\rho _{20}, \end{aligned}$$

14 $$\begin{aligned} {\dot{\rho }}_{33}&=\gamma _{33}\rho _{00}-\sum _{m=1,2}^{3}\gamma _{m3}\rho _{33}-i\Omega _{p_{2}}\rho _{03}+i\Omega _{p_{2}}^{*}\rho _{30}, \end{aligned}$$

15 $$\begin{aligned} {\dot{\rho }}_{10}&=i{\mathcal {L}}_{10}\rho _{10}-i\Omega _{p_{1}}\rho _{00}+i\Omega _{p_{1}}\rho _{11}+i\Omega _{c}\rho _{12}+i\Omega _{p_{2}}\rho _{13}, \end{aligned}$$

16 $$\begin{aligned} {\dot{\rho }}_{20}&=i{\mathcal {L}}_{20}\rho _{20}-i\Omega _{c}\rho _{00}+i\Omega _{c}\rho _{22}+i\Omega _{p_{1}}\rho _{21}+i\Omega _{p_{2}}\rho _{23}, \end{aligned}$$

17 $$\begin{aligned} {\dot{\rho }}_{30}&=i{\mathcal {L}}_{30}\rho _{30}-i\Omega _{p_{2}}\rho _{00}+i\Omega _{p_{2}}\rho _{33}+i\Omega _{p_{1}}\rho _{31}+i\Omega _{c}\rho _{32}, \end{aligned}$$

18 $$\begin{aligned} {\dot{\rho }}_{12}&=i{\mathcal {L}}_{12}\rho _{12}-i\Omega _{p_{1}}\rho _{02}+i\Omega _{c}^{*}\rho _{10}, \end{aligned}$$

19 $$\begin{aligned} {\dot{\rho }}_{13}&=i{\mathcal {L}}_{13}\rho _{13}-i\Omega _{p_{1}}\rho _{03}+i\Omega _{p_{2}}^{*}\rho _{10}, \end{aligned}$$

20 $$\begin{aligned} {\dot{\rho }}_{23}&=i{\mathcal {L}}_{23}\rho _{23}-i\Omega _{c}\rho _{03}+i\Omega _{p_{2}}^{*}\rho _{20}, \end{aligned}$$

constrained by $\rho _{11}+\rho _{22}+\rho _{33}=1$ and $\rho _{ij}=\rho _{ji}^{*}$.

Here, $${\mathcal {L}}_{10}=\delta _{p_{1}}+\delta _{B}+i\gamma _{10}$$, $${\mathcal {L}}_{20}=\delta _{c}+i\gamma _{20}$$, $${\mathcal {L}}_{30}=\delta _{p_{2}}-\delta _{B}+i\gamma _{30}$$, $${\mathcal {L}}_{12}=\delta _{c}-\delta _{p_{1}}-\delta _{B}-i\gamma _{12}$$, $${\mathcal {L}}_{13}=\delta _{p_{2}}-\delta _{p_{1}}-2\delta _{B}-i\gamma _{13}$$, $${\mathcal {L}}_{23}=\delta _{p_{2}}-\delta _{B}-\delta _{c}-i\gamma _{23}.$$

### Induced torque in the linear regime

The steady-state solutions to the optical Bloch equations are considered in our tripod model. When the intensity of the control field is stronger than the intensity of both probe fields, the stationary population distribution is symmetric with respect to $1\leftrightarrow 3$ exchange. This allows the atoms to be initially populated in lower levels $|1\rangle$ and $|3\rangle$, with the population of the other two levels vanishing. Keeping the lowest order contribution in the probe fields, the analytical solutions for the off-diagonal (linear) density matrix $\rho _{10}$ and $\rho _{30}$ read then

21 $$\begin{aligned} \rho _{10}=\frac{1}{2}\frac{\Omega _{p_{1}}{\mathcal {L}}_{12}}{{\mathcal {L}}_{10}{\mathcal {L}}_{12}-|\Omega _{c}|^{2}}, \end{aligned}$$

22 $$\begin{aligned} \rho _{30}=\frac{1}{2}\frac{\Omega _{p_{2}}{\mathcal {L}}_{23}^{*}}{{\mathcal {L}}_{30}{\mathcal {L}}_{23}^{*}-|\Omega _{c}|^{2}}. \end{aligned}$$

The torque function, which characterizes the induced torque on the four-level tripod atom system, is determined by the density matrix elements $\rho _{10}$ and $\rho _{30}$, as outlined in Eq. (9). To investigate the linear regime, we make specific assumptions in order to derive expressions for the linear density matrix elements $\rho _{10}$ and $\rho _{30}$, as presented in Eqs. (21), (22). In the linear regime, we assumed that the intensity of the probe beams and is significantly weaker than that of the control field ( $\Omega _{p_{1}},\Omega _{p_{2}}<<\Omega _{c}$). Additionally, we considered the atom to be initially in the lower states $|1\rangle$ and $|3\rangle$ ($\rho _{11}=\rho _{33}=1/2$), with the population of the other two levels vanishing. By expanding the density matrix equations to the first order of the probe fields, we obtained the expressions in (21), (22), enabling to control the torque function in the linear regime.

The torque function given in Eq. (9) is dependent on the system parameters, which can be seen from Eqs. (10), (21), (22). Additionally, the torque depends on the position-dependent Rabi frequencies $\Omega _{p_{1}}$ and $\Omega _{p_{2}}$ (LG beams) given that the tripod atoms are trapped in the ring of a LG beam. However, since the Rabi frequencies of probe fields are almost constant in this region, the plots presented in this paper are valid for $g_{p_{1}}$and $g_{p_{2}}$ to a very good approximation^41^. For the simulations in the linear regime in this paper, we assume $g_{p_{1}}=g_{p_{2}}=0.1\gamma$ and plot all the results with respect to $\delta _{p_{1}}=\delta _{p_{2}}=\delta _{p}$. We also assume that $\gamma _{10}=\gamma _{20}=\gamma _{30}=\gamma$, and all the system parameters are scaled by $\gamma$. Consistent with the scaling used for other parameters, the Zeeman shift is also scaled by $\gamma$. To investigate the effect of changing the magnetic field *B* on the induced torque, we introduce a scaling factor $\Gamma$, given by $\Gamma =\hbar \mu _{B}^{-1}g_{s}^{-1}B$^46^. This scaling enables us to examine the effects of varying magnetic field strengths without altering the other system parameters.

**Figure 2:**
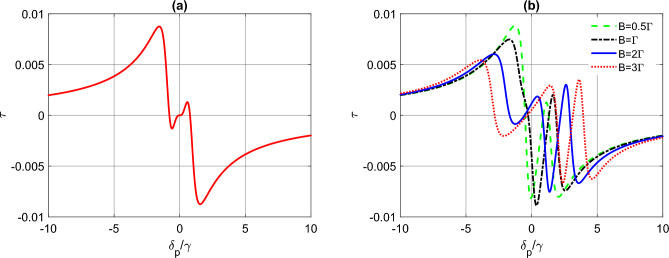
The induced torque function $\tau$ (in units of $\textrm{s}^{-1}$) as a function of detuning $\delta _{p}$ (in units of $\gamma$), for a tripod atom system. The parameters used are: $g_{p_{1}}=g_{p_{2}}=0.1\gamma$, $\Omega _{c}=\gamma$, $\delta _{c}=0$, $\gamma _{10}=\gamma _{20}=\gamma _{30}=\gamma$, $\gamma _{12}=\gamma _{23}=0.1\gamma$, and (**a**) $B=0$, and (**b**) for different values of *B*.

We begin by investigating the influence of magnetic field on the induced torque experienced by tripod atoms, and explore the potential for using this parameter to control the torque. Figure 2 depicts the variation of torque against the probe detuning, in the absence of dephasing, for different values of magnetic field strength. In Fig. 2a, we consider the resonant case without magnetic field ($B=0$) and with a control field intensity of $\Omega _{c}=\gamma$. Here, the torque exhibits a dispersive behavior with a positive slope around the line center, and vanishes at $\delta _{p}=0$. The torque has a positive value for negative detunings, and a negative value for positive detunings. This case corresponds to the appearance of EIT in the tripod scheme, where the system can be seen as two adjacent $\Lambda$ systems, each involving one of the probe fields, and sharing the same control field^45^.

When the magnetic field is turned on (Fig. 2b), the dispersive torque shifts towards the right, and becomes nonzero at zero probe detuning. In this case, the torque acquires a positive value for the right channel of probe detuning. However, the magnitude of the torque remains almost unchanged when compared to the $B=0$ case. This demonstrate the potential of magnetic field as a control parameter to modify the induced torque on the tripod atoms.

**Figure 3:**
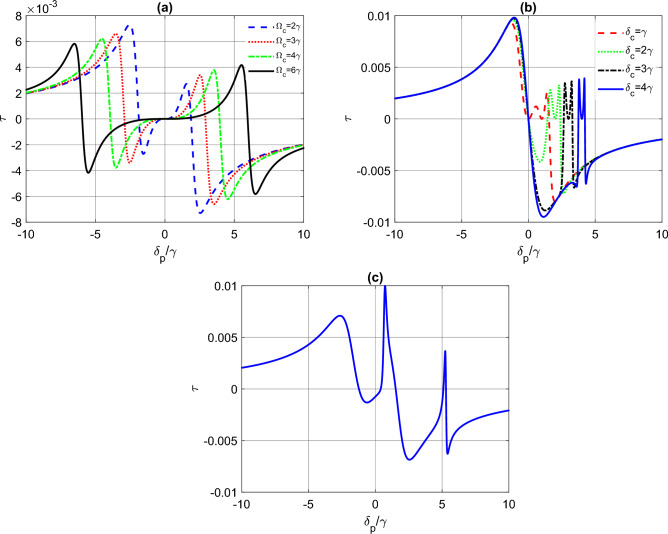
The induced torque function $\tau$ (in units of $\textrm{s}^{-1}$) as a function of detuning $\delta _{p}$ (in units of $\gamma$) for a tripod atom system. In (**a**) $B=0$, $\delta _{c}=0$ and $\Omega _{c}$ varries. In (**b**) $B=0$, $\Omega _{c}=\gamma$ and $\delta _{c}$ varries. In (**c**), $\Omega _{c}=\gamma$, $\delta _{c}=3\gamma$ and $B=2\Gamma$. The other parameters are the same as Fig. 2.

In addition to the influence of the magnetic field on the variation of induced torque on tripod atoms, we also investigate the impact of control field intensity and detuning on the torque. Figure 3a shows the effect of increasing the control field intensity while keeping the control field detuning at resonance ($\delta _{c}=0$). As we can see, increasing $\Omega _{c}$ leads to the appearance of nonzero torque for larger probe detunings. In Fig. 3b, we study the effect of control field detuning for a fixed control field intensity ($\Omega _{c}=\gamma$) with the magnetic field switched off. Here, a twin peaks model appears in the right channel as we increase the control field detuning. In both cases, the torque is zero at zero probe detuning. To further illustrate the impact of the magnetic field, we set $\Omega _{c}=\gamma$ and $\delta _{c}=3\gamma$ and turn on the magnetic field with $B=2\Gamma$ (Fig. 3c). In this case, the torque shows two sharp peaks with the peaks well seperated compared to Fig. 3b.

**Figure 4:**
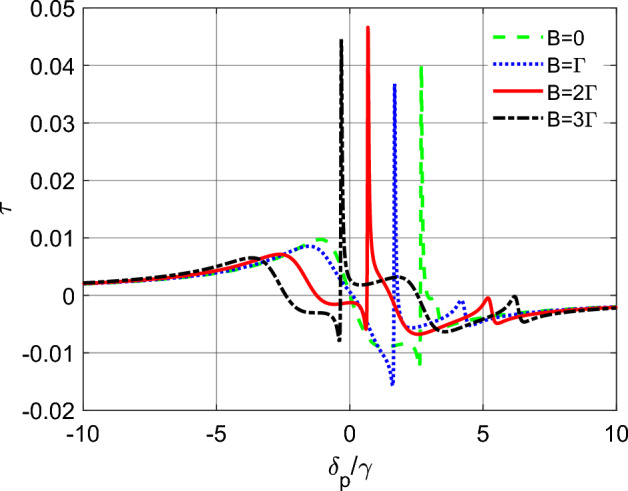
The induced torque function $\tau$ (in units of $\textrm{s}^{-1}$) as a function of detuning $\delta _{p}$ (in units of $\gamma$) for a tripod atom system and for different values of *B*. Here, $\Omega _{c}=\gamma$, $\delta _{c}=3\gamma$, $\gamma _{12}=\gamma _{23}=0.1\gamma$ and the other parameters are the same as Fig. 2.

The magnitude of torque can be significantly enhanced when the dephasing terms are non-zero, as shown in Fig. 4. When the dephasing rates $\gamma _{12}$ and $\gamma _{23}$ are equal to $0.1\gamma$, the torque exhibits a resonant-like behavior and reaches a maximum that is larger than the previous cases where the dephasing was neglected. Interestingly, the maximum torque can be well-controlled by varying the magnetic field strength *B*. Hence, adjusting the Zeeman shift of atom levels, which is induced in the presence of a magnetic field, can efficiently manipulate the torque, allowing for enhanced torque at specific frequency detunings while achieving vanishing torque at other detunings. This observation suggests a simple and efficient way to control the rotational motion of a tripod atom system. By modulating the magnetic field, it is possible to manipulate the atom’s magnetic moment and its coupling to the external fields, thereby affecting the induced torque. In addition, the dephasing terms lead to a manipulation of the resonance peaks and a modification of the torque shape. This implies that the torque can be tuned to respond differently to changes in the probe detuning and the control field intensity or detuning, allowing for greater control of the system’s rotational motion. This highlights the potential of using magnetic fields to manipulate the rotational dynamics of tripod atom systems, which could have significant implications for applications in areas such as quantum information processing and precision sensing. In what follows we explore a more complex condition (when nonlinearities are present) to maximize the torque and achieve greater control over the system’s motion.

### Higher-order contributions on the induced torque

The expressions for the density matrix elements, $\rho _{10}$ and $\rho _{30}$, presented in Eqs. (21), (22) were obtained by considering only the lowest first-order contribution of the probe fields in the optical susceptibility of the medium. This simplification was made because the intensity of the probe fields was much weaker than the control field, in the previous section where the induced torque was manipulated.

However, in this section, we investigate the behavior of the induced torque on the tripod atoms when the probe pulses are not necessarily weak. To achieve this, we derive general expressions for the steady-state probe field density matrix elements, $\rho _{10}$ and $\rho _{30}$, including all higher-order contributions of the probe fields. In particular, we obtain the steady-state solutions to the density matrix equations by considering the two lowest order contributions in probe fields, which encompassed linear and third-order nonlinear susceptibilities.These expressions can be written as^45^

23 $$\begin{aligned} \rho _{10}=-\frac{1}{2}\lambda _{1}\Omega _{p_{1}}\left( \frac{{\mathcal {L}}_{12}{\mathcal {L}}_{13}}{{\mathcal {L}}_{10}{\mathcal {L}}_{12}{\mathcal {L}}_{13}-{\mathcal {L}}_{13}\Omega _{c}^{2} -{\mathcal {L}}_{12}\Omega _{p_{2}}^{2}}+\frac{{\mathcal {L}}_{12}{\mathcal {L}}_{13}{\mathcal {L}}_{23}\Omega _{p_{2}}^{2}}{{\mathcal {L}}_{30}^{*}{\mathcal {L}}_{13}{\mathcal {L}}_{23}-{\mathcal {L}}_{13}\Omega _{c}^{2} -{\mathcal {L}}_{23}\Omega _{p_{1}}^{2}}\right) , \end{aligned}$$

24 $$\begin{aligned} \rho _{30}=-\frac{1}{2}\lambda _{2}\Omega _{p_{2}}\left( \frac{{\mathcal {L}}_{23}^{*} {\mathcal {L}}_{13}^{*}}{{\mathcal {L}}_{30}{\mathcal {L}}_{13}^{*}{\mathcal {L}}_{23}^{*} -{\mathcal {L}}_{13}^{*}\Omega _{c}^{2}-{\mathcal {L}}_{23}^{*}\Omega _{p_{1}}^{2}} +\frac{{\mathcal {L}}_{12}^{*}{\mathcal {L}}_{13}^{*}{\mathcal {L}}_{23}^{*}\Omega _{p_{1}}^{2}}{{\mathcal {L}}_{10}^{*}{\mathcal {L}}_{12}^{*}{\mathcal {L}}_{13}^{*}-{\mathcal {L}}_{13}^{*}\Omega _{c}^{2} -{\mathcal {L}}_{12}^{*}\Omega _{p_{2}}^{2}}\right) , \end{aligned}$$

where

25 $$\begin{aligned} \lambda _{1}&=\frac{1}{1+\frac{\frac{{\mathcal {L}}_{12}{\mathcal {L}}_{23}}{{\mathcal {L}}_{13}^{2}}\Omega _{p_{1}}^{2}\Omega _{p_{2}}^{2}}{4({\mathcal {L}}_{10}{\mathcal {L}}_{12} -\Omega _{c}^{2})({\mathcal {L}}_{30}^{*}{\mathcal {L}}_{20}-\Omega _{c}^{2})}}, \end{aligned}$$

26 $$\begin{aligned} \lambda _{2}&=\frac{1}{1+\frac{\frac{{\mathcal {L}}_{23}^{*}{\mathcal {L}}_{12}^{*}}{{\mathcal {L}}_{13}^{*}} \Omega _{p_{1}}^{2}\Omega _{p_{2}}^{2}}{4({\mathcal {L}}_{30}{\mathcal {L}}_{23}^{*} -\Omega _{c}^{2})({\mathcal {L}}_{10}^{*}{\mathcal {L}}_{12}^{*}-\Omega _{c}^{2})}}. \end{aligned}$$

In the linear regime ($\Omega _{p_{1}},\Omega _{p_{2}}<<\Omega _{c}$), the equations for the density matrix elements, $\rho _{10}$ and $\rho _{30}$, reduce to those presented in Eqs. (21), (22). However, to study the torque variations in the nonlinear regime where the intensity of the probe fields is large, we need to replace Eqs. (23), (24) into Eq. (9).

In this section, we consider a scenario where the intensity of the probe fields is strong enough to be comparable with the intensity of the control fields. Specifically, we set $g_{p_{1}}=g_{p_{2}}=\Omega _{c}=\gamma$ and investigate the effect of the magnetic field on the induced torque variations. It is important to note that in the absence of dephasing, the density matrix elements, $\rho _{10}$ and $\rho _{30}$, which contain now nonlinear contributions, have a singularity on resonance of the optical fields. Hence, to avoid this singularity in our simulations, we introduce a non-zero dephasing term, $\gamma _{13}=\gamma _{12}=\gamma _{23}=0.1\gamma$.

**Figure 5:**
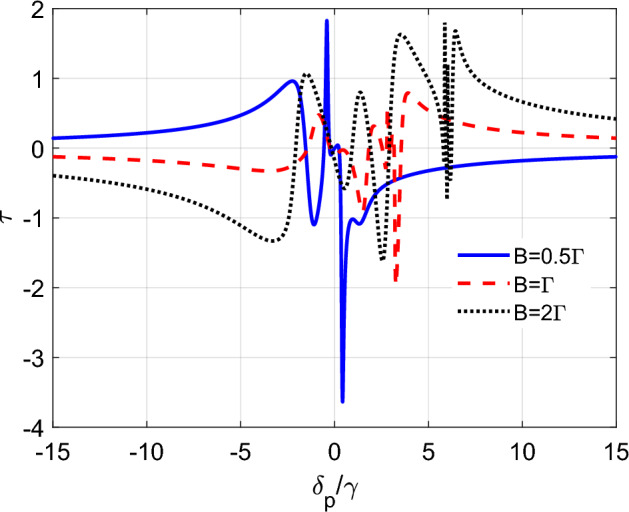
The induced torque function $\tau$ (in units of $\textrm{s}^{-1}$) as a function of detuning $\delta _{p}$ (in units of $\gamma$) for a tripod atom system and for different values of *B*. Here $g_{p_{1}}=g_{p_{2}}=\Omega _{c}=\gamma$, $\gamma _{13}=\gamma _{12}=\gamma _{23}=0.1\gamma$ and the other parameters are the same as Fig. 2.

Figure 5 illustrates the variation of the induced torque for intense probe pulses, as well as the effect of the magnetic field on the induced torque in this regime. As shown in the figure, the magnitude of the induced torque is significantly enhanced compared to the linear regime (as observed in Figs. 2, 3, 4). When the intensity of the probe field is weak, the induced torque only has a linear contribution, due to the non-zero stationary population in levels $|1\rangle$ and $|3\rangle$. However, in the current scenario where the intensity of the probe field is strong, the induced torque is also affected by higher-order terms, such as the cross-Kerr nonlinearity contributions, resulting in an enhanced torque effect. Manipulating the magnetic field introduces additional sharp peaks in the torque diagram, shifting the maximum to different probe detunings. This behavior can be explained by the presence of additional energy levels in the atom’s structure that are sensitive to the magnetic field. Hence, by adjusting the magnetic field, we can tune the induced torque at different probe detunings, offering more flexibility in the manipulation of the atom’s motion.

## Conclusions

In conclusion, this study delved into the manipulation of induced torque in a four-level tripod atom system interacting with two vortex probe beams and a non-vortex control beam. In the linear regime when the probe vortex beams are much weaker than the contol field, we demonstrated effective control over the induced torque by fine-tuning parameters such as magnetic field strength, control field intensity, detuning, and dephasing terms. Our analysis highlighted the significant role of the Zeeman shift-induced magnetic field, which not only enhanced the torque but also exhibited a distinct peak behavior concerning probe detuning, allowing for the precise control of rotational motion within the system. Furthermore, our investigation extended to the nonlinear regime, where the intensity of the probe fields approached that of the control field. In this regime, we observed higher-order contributions to the induced torque, leading to further enhancement of the light induced torque. This opens up possibilities for even greater control and manipulation of atomic rotational dynamics. The ability to manipulate induced torque in tripod atoms systems holds promise for a range of applications, including precision measurement, quantum information processing, and quantum control. With improved control over rotational motion, we can explore advanced technologies and harness the potential of these systems for future advancements.

## Data Availability

Te datasets used and/or analyzed during the current study available from the corresponding author on reasonable request.
